# Occupational Therapy Practice in Sleep Management: A Review of Conceptual Models and Research Evidence

**DOI:** 10.1155/2018/8637498

**Published:** 2018-07-29

**Authors:** Eris C. M. Ho, Andrew M. H. Siu

**Affiliations:** ^1^Occupational Therapy Department, Queen Elizabeth Hospital, Hospital Authority, Kowloon, Hong Kong; ^2^Department of Rehabilitation Sciences, The Hong Kong Polytechnic University, Kowloon, Hong Kong

## Abstract

The effectiveness of sleep intervention developed by occupational therapists was reviewed, and a conceptual framework for organizing the developing practice of sleep management in occupational therapy was proposed in this paper. Evidence-based articles on sleep management practice in occupational therapy from 2007 to 2017 were retrieved. Four types of effective sleep management intervention were identified from the literature, including the use of assistive devices/equipment, activities, cognitive behavioral therapy for insomnia, and lifestyle intervention, and the use of assistive device was the most popular intervention. Applying the Person-Environment-Occupation Performance (PEOP) framework, we developed a conceptual framework for organizing occupational therapy practice in sleep management. The future development of occupation-based sleep intervention could focus on strategies to (1) minimize the influence of bodily function on sleep, (2) promote environment conducive to sleep, and (3) restructure daytime activity with a focus on occupational balance.

## 1. Introduction

Sleep problem is the difficulty in initiating or maintaining sleep or suffering from nonrestorative sleep accompanied by daytime functional impairment [1]. Sleep problems are a worldwide health issue, with an average prevalence rate ranging from 10% to 30% in developed countries [2, 3]. Hong Kong, a fast-paced city, has a relatively high prevalence (39.4%) of sleep problems [4]. Sleep is important for health and well-being. People with sleep problems are prone to suffer from serious medical conditions, such as obesity, heart disease, high blood pressure, and diabetes [5]. Sleep problems also affect cognitive performance, including alertness, reaction, memory, and learning [6]. Very often, sleep problems could impact on daily occupations such as work, daily activities, social performance, and well-being.

It is uncommon for people to seek help for insomnia [7, 8]. Instead of consulting health care professionals, many people use over-the-counter sleeping pills or use alcohol or substances to cope with sleep problems. Both methods can offer only a temporary or limited improvement of sleep quality, and the addictive and side effects may pose significant threats to health and well-being in the long run. In general, health care management of sleep problems involves pharmacological and/or nonpharmacological interventions [9]. Pharmacological intervention should be monitored by physicians, and medication is usually prescribed on a short-term basis, given the concerns of potential dependence and the side effects of medication on cognitive performance. Nonpharmacological sleep interventions often include sleep hygiene education, relaxation [10, 11], and cognitive-behavioral treatment for insomnia which targets the modification of maladaptive thoughts that perpetuate insomnia [12].

In occupational therapy theories, sleep is conceptualized as a restorative occupation with the goal of rest and recuperation, and good sleep and rest could support the formation of the occupation mix of self-care, work, and leisure during the day [13–15]. The concepts of occupational balance focus on time use and suggest that the balance between rest/sleep and daytime activity is important in promoting function and well-being [13, 16–18]. Sleep has a significant impact on functional performance in self-care, work, and leisure. Thus, sleep and daytime functioning are closely interrelated, and excessive or insufficient sleep or daytime activities will contribute to occupational imbalance.

As a member of the primary care team, there is clearly a growing need for occupational therapists to provide interventions for patients with sleep problems and related mental health issues. To facilitate the development of sleep management practice in occupational therapy, there is a need to further conceptualize on how sleep and occupation are linked and identify evidence-based occupational-based interventions that could be used in clinical practice.

In sum, only a few conceptual models or frameworks, like the PEOP model or the concept of occupational balance, have attempted to discuss how sleep is related to occupation, and how it could fit into occupational therapy practice. While some evidence-based studies have been published on sleep interventions in occupational therapy practice, there appears to be great diversity in the target groups, objectives, and components of programs, and/or in methodology. There has been no formal review or analysis of the characteristics of intervention programs or their findings. This article aims to conduct a systematic review of the literature on occupational therapy theories and practice for patients with sleep problems and the research evidence published in the past ten years. The objectives are to (1) identify the key intervention approaches and the components of sleep management administered by occupational therapists, (2) examine the research evidence on the effectiveness of occupational therapy interventions for people with sleep problems, and (3) formulate a conceptual framework for sleep management in occupational therapy.

## 2. Review of Research Evidence

### 2.1. Literature Search

**Figure 1:**
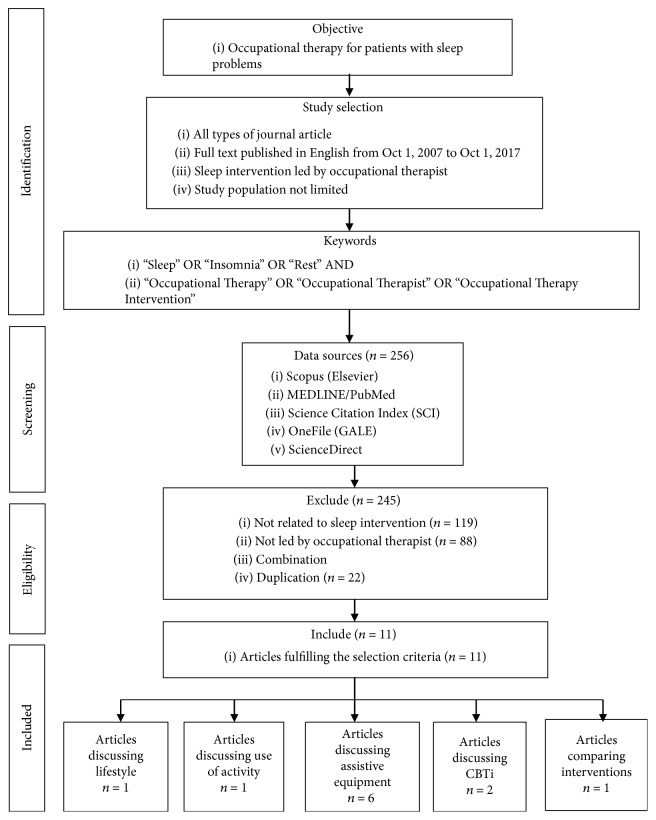
Flowchart of the literature search and selection process.

A literature search was performed according to the 2009 PRISMA Statement for systematic reviews [19]. Two researchers performed the review using the OneSearch search engine of the Hong Kong Polytechnic University, which integrated a number of research databases. The inclusion criteria were (1) sleep intervention developed by an occupational therapist, (2) sleep as primary outcome, (3) peer-reviewed articles, and (4) written in English. Papers only describing the role of occupational therapists in sleep management, theoretical papers, books, and editorial were excluded. The search included papers published between October 1, 2007, and October 1, 2017 (the previous ten years to the review). To ensure a comprehensive coverage of the literature, the search terms included “sleep,” “rest,” or “insomnia” and “occupational therapy,” “occupational therapist,” or “occupational therapy intervention.” Keyword searches were performed in five key databases: Scopus (Elsevier), MEDLINE/PubMed, Science Citation Index (SCI), OneFile (GALE), and ScienceDirect. 256 articles were included, and after applying inclusion and exclusion criteria, 11 articles were retained for data synthesis [20–30].  Figure 1 shows the systematic search and review process.

### 2.2. Risk of Bias

Two assessors conducted the quality assessment of the articles. Level of evidence [31] was graded based on the research design. Eight out of the eleven articles were categorized as level III or above in terms of evidence, which indicates well-conducted studies with strong evidence including consistent results (Table 1). Three articles involved randomized controlled trials (RCT). Based on the *Cochrane Handbook of Reviews of Effectiveness of Interventions*, we evaluated the risk of bias in the three RCT studies (Table 2). The result showed that allocation sequence generation, concealment, and blinding were adequately performed in two out of the three studies. However, there are variations in detection and attrition bias among them. The design of the RCT studies are quite different: (1) there is broad variation in the duration of intervention (varied from three days to six months); (2) study samples include both adults and elderly; and (3) there is a lack of detail on the research methodology, for example, regarding randomization, baseline measurements, and blinding procedures. As the number of studies is small and there is inadequate information to conduct a formal meta-analysis of the interventions, a descriptive approach was used in the review of the research evidence.

Mixed Methods Appraisal Tool (MMAT) 2001 version [32] is adopted for quality assessment. This is a five-item checklist, designed for assessing the quality of the articles in relation to different types of research designs. Four areas focus on the methodology, outcomes, statistical process, and result interruption. Each item scored 0 or 1 and yield a maximum of 4 points (100%). The quality of 11 articles was examined, and the results are shown in Table 1. Overall speaking, 55% of the articles demonstrate satisfactory quality with rating 3/4 (75%) in MMAT and all level I and level II studies achieved satisfactory rating.

## 3. Analysis of Research Evidence

On the whole, occupational therapy-based sleep intervention was found to be effective in improving patients' sleep to different extents. Coverage of existing services and types of intervention were reviewed systematically to build up knowledge for further discussion. Three of the studies were level I RCTs [21, 23, 26], one was a level II nonrandomized study [25], three were level III one-group nonrandomized pretest and posttest studies (Eakman et al. 2016; [24, 30]), two were level IV descriptive studies that included analysis of outcomes (case series) [28, 29], and one was a level V case study [22]. Ten studies were from the United States, and one from Canada; no study on sleep intervention was published in an Asian country during the review period.

### 3.1. Characteristics of Target Population

To conduct an analysis of the articles, we summarized the study background (author, year of publication, and country), format (design and sample size), patients' characteristics (age range, sex, setting, and type of disease), and type of occupational therapy outcome and intervention (Table 3).

Sample size varies from two [28, 30] to 217 [26], and participants' age ranges from 30 days to 82 years old. The influence of participant group in sleep management is diverse. The diagnoses of participants include people with an autistic spectrum disorder (ASD) [22, 27, 29, 30], traumatic brain injury (TBI) [28], and posttraumatic stress disorder (PTSD) (Eakman et al. 2016). Other study participants include community-dwelling elderly, adults with sleep problems [23, 26], and in-patients [21, 24, 25]. Two studies focus on people with primary insomnia who had no medical or psychiatric problems, and whose insomnia was likely related to lifestyle and aging issues [23, 26].

### 3.2. Key Types of Sleep Intervention and Effectiveness

Four types of sleep intervention were identified: (1) use of assistive devices/equipment [21, 22, 24, 25, 27, 29]; (2) use of activities [28]; (3) cognitive behavioral therapy for insomnia (Eakman et al. 2016; [30]); and (4) lifestyle intervention [26]. Gutman et al. [23] compared the effectiveness of three different interventions: sleep aids, meditation activity, and sleep hygiene.

#### 3.2.1. Use of Assistive Device/Equipment

Environment can significantly affect one's sleep, and a key occupational therapy intervention is the use of assistive aids or positioning to facilitate sleep onset. The six articles that used sleep aids evaluated the effectiveness of the Dreampad pillow, weighted blankets, and sleep tools including eye masks, earplugs, and white noise machines. The Dreampad pillow is a patented technology which conducts soothing music in the pillow that relaxes the body and mind, and it is supported with a music app of library of research-backed sleep-inducing music. The studies on the Dreampad pillow show that it could significantly improve sleep duration and latency [27, 29], improve sleep quality, and reduce nighttime awakenings [23]. It also helps to improve secondary outcomes, including autism-related behaviors and attention [27, 29] and quality of life and parent satisfaction (Schoen et al. 2016). Gee and colleagues (2007) found that the weighted blanket, a sleep aid developed for patients with ASD, could increase sleep duration and shorten latency.

Overall, there is much evidence supporting the effectiveness of sleep aids in promoting patients' sleep and reducing sleep disturbance during hospital stays [24] or reducing physical symptoms like pain [21] and fatigue [24]. Other than prescribing aids, there are also studies on how positioning could promote sleep in preterm babies. Jarus and colleagues [25] found that prone position showed more sleep pattern and less awake patterns than supine position.

#### 3.2.2. Use of Activities

Two studies use mind-body activities to promote sleep, including iRest meditation, yoga, and breathing [23, 28]. It is generally believed that calming or mindful activity can improve sleep quality at night, but effectiveness varies in the articles reviewed. The use of meditation activity was found to result in statistically longer sleep time than sleep hygiene education alone [23]. Yoga and breathing techniques were not found to increase sleep duration, but could reduce depressive symptoms [28].

#### 3.2.3. Cognitive Behavioral Therapy for Insomnia (CBTi)

In recent years, increasing numbers of occupational therapists have undergone training in how to conduct CBTi for patients with sleep problems. CBTi is a structured program which aims to improve sleep by identifying and changing the negative thoughts and behaviors related to it, such as cognitive traps and beliefs concerning sleep restriction [10, 12]. CBTi is usually conducted on a weekly basis and monitored via different assessments, like a sleep diary. Two of the studies adopted CBTi as the core of their sleep management program. They found that CBTi could significantly improve the ability to handle sleep issues in patients with PTSD [30], reduce their sleeping difficulties and nightmares, reduce dysfunctional sleep beliefs, and improve their ability to participate in social roles (Eakman et al. 2016).

#### 3.2.4. Lifestyle Intervention

Among the selected articles, there is one large-scale RCT that focuses on lifestyle intervention to promote sleep among community-dwelling elderly [26]. The lifestyle intervention emphasizes the promotion of healthy sleep habits and activity rescheduling and facilitates role transition in aging through education, experience sharing, and goal setting. Too much or too little daytime activity is highly related to sleep pattern at night; rescheduling of daytime activity helps one in achieving a balanced lifestyle to facilitate sleep during night time. The program demonstrates positive changes in sleep behaviors, including increased sleeping hours, reduced sleep difficulties, and reduced nightmares [26]. Clients also reduced daytime napping and increased daytime engagement, especially social activities. This study suggests that sleep management does not only just concern sleep but also daytime functioning.

## 4. Conceptual Framework for Occupational Therapy Practice in Sleep Management

**Figure 2:**
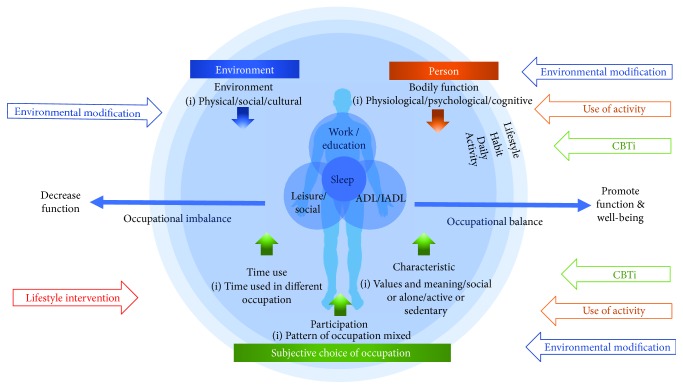
Occupational therapy on sleep management.

Few of the 11 articles explicitly mention the conceptual or theoretical framework used. Only two studies mention the use of cognitive behavioral therapy as a framework for guiding practice (Eakman et al. 2016; Wooster et al. 2016), while another refers to daytime engagement and how it is related to sleep based on a lifestyle redesign program [26]. In this part of the review, we would like to propose a conceptual model for organizing sleep interventions and occupational therapy practice based on the PEOP framework (Christiansen et al. 2011).  Figure 2 shows how we could integrate the theory and practice of occupational therapy based on current research evidence and explains the unique role of occupational therapy in sleep management.

Based on the PEOP framework, occupation-based sleep management can focus on three levels: (1) person: minimizing the influence of bodily function on sleep (Eakman et al. 2016; [22, 25, 28, 30]); (2) environment: promoting environment conducive to sleep [21, 24, 27, 29]; and (3) occupation: restructuring daytime activity [26].

First, the “person” level relates to bodily function, which includes physiological, psychological, and cognition performance. Bodily function can affect one's sleep. People suffering from depression and pain and the elderly very often have sleep problems (Foley et al. 2004). Sleep interventions targeting bodily function could include the use of activity to promote calming effects on the body to shorten sleep latency [23, 28]; CBTi could be applied to manage the cognitive traps in PTSD (Eakman et al. 2016; [30]); or a weighted blanket could be used to address the overresponsivity of children with ASD [22] or to position a preterm baby [25]. Although bodily function cannot be improved quickly and is not totally reversible, this factor should also be considered with the aim of maximizing functioning.

Second, occupational therapists could use environmental interventions in the physical, social, or cultural domains to address sleep problems. This review shows that the control of environmental factors plays a significant role in sleep management [21, 24, 27, 29]. Examples of such an intervention include the use of the newly designed Dreampad pillow, weighted blankets, and sleep tools; controlling light, temperature, and humidity levels; and the use of body positions to tackle sleep problems caused by specific diseases. In Hong Kong, a densely populated city, living environment may create sleep barriers for patients. Circumstances permitting the use of sleep tools to reduce environmental stimuli may facilitate sleep. Besides physical environment, social environmental factors, such as sleep partner (both human and pets), also affect sleep and should be considered in an intervention.

Third, the subjective choice of daily activities is the most important area in future service development for sleep management. Everyone has the right to determine the combination of daily occupations to achieve occupational balance even when suffering from illness. However, one's daytime activity can clearly have an impact on one's sleep [26]. In sleep management programs, it is important for therapists to guide clients to choose daily activities and develop occupational balance (Figure 1), including how to organize daytime occupations (activity of daily living/household, work/education, and leisure/social), how to allocate time to daily occupations, and how to restructure activity patterns according to the meaning and purpose of activities.

The above analysis shows that occupational therapists provide sleep management guidance to patients from diverse disease groups in all age groups. Most of the programs presented in the literature were developed for a specific disease group, such as children with ASD ([22, 27, 29]; Wooster et al. 2016) or traumatic brain injury [28]. Although sleep is highly correlated with mental condition, the development of sleep programs in mental healthcare is relatively limited. Only one study among the 11 reviewed articles focuses on this (Eakman et al. 2016). The role of occupational therapists in sleep management in mental health settings has been explored over a long time (Faulkner et al. 2015), but the effectiveness of the proposed intervention has not been evaluated scientifically. A huge number of people suffering from insomnia in Hong Kong present with initial mood disturbance; early intervention plays an important role to prevent long-term healthcare burden.

## 5. Occupational Therapy-Based Sleep/Daytime Functional Assessment

This review has shown that occupational therapists should assess the occupational balance between daytime activities and sleep. Limited information on occupational therapy-related assessment was found in the literature. The Canadian Occupational Performance Measurement (COPM) (Eakman et al. 2016; [21]) and the Functional Independent Measure (FIM) [21] have been used in some sleep management studies to investigate daytime functioning. FIM focuses on one's functioning while COPM focuses on occupational performance and satisfaction, without fully exploring lifestyle. Dür et al. [33] developed an occupational balance questionnaire (OB-Quest) to further explore occupational balance, and it can be applied to assess the occupational balance of both patients and healthy individuals. More comprehensive occupation-based assessment will definitely advance the development of sleep management in occupational therapy.

## 6. Limitations

There are two key limitations of this study. First, only eleven studies were identified by the review. Although this reflects the lack of evidence-based studies of sleep intervention in occupational therapy, we may consider expanding the search and be more inclusive in the review. One such possibility is to widen the search criteria and include papers studying multidisciplinary sleep intervention programs in which the occupational therapists participate as a team member. The second limitation of this study is that it could not provide estimates on effect sizes of occupational therapy interventions in sleep management. Only three studies are level I clinical trials which could be used for meta-analysis. We decided not to proceed with meta-analysis based on a small number of studies, which are implemented using different intervention methods for different clients.

While studies on sleep in occupational therapy have become increasingly conspicuous, the number of relevant literature remains limited. It is necessary to extend the scope of research by including patients from different disease groups and types and patients with reported history of sleep problems. There is a great diversity in the methodologies adopted by the selected articles, including case-control trial, RCT, and case series. If only RCTs were to be reviewed here, only three articles would have been included, which would have limited the analysis. The heterogeneity of study designs may also have affected the analysis. Moreover, specific database in occupational therapy may be considered to include for revealing more related articles in occupational therapy such as OTseeker and OTsearch.

## 7. Conclusion

Sleep is a restorative occupation from the occupational therapy perspective. Its main function is to help us recover from daytime occupations, to build up energy to move forward. The selected literature provides an overview of the scope and types of sleep intervention. The findings, along with the rising incidence of sleep problems, indicate a need for further exploration in this topic. Occupational therapists could address the needs of people with insomnia, by developing sleep management programs using environmental intervention, assistive devices/equipment, the use of activity, CBTi, and lifestyle interventions. Based on the PEOP framework, occupation-based sleep interventions can aim to (1) minimize the influence of bodily function on sleep; (2) promote environment conducive to sleep; and (3) restructure daytime activity with a focus on occupational balance. Further development of sleep management from an occupational therapy perspective will strengthen the role of sleep within clinical practice, education, and research domains.

## Figures and Tables

**Table 1:** Quality assessment by Mixed Methods Appraisal Tool (MMAT) 2001 version.

Author, year	Research design	Type	Level of evidence (LoE)	Quality appraisal (MMAT)
Eakman et al., 2016	Single-arm feasibility pilot study	Quantitative descriptive	Level III	3/4
Farrehi et al., 2016 [21]	RCT	Quantitative randomization controlled (trials)	Level I	3/4
Gee et al., 2017 [22]	An ABA single-subject design	Qualitative	Level V	1/4
Gutman et al., 2016 [23]	RCT	Quantitative randomization controlled (trials)	Level I	3/4
Heidt et al., 2016 [24]	Experimental study design	Quantitative descriptive	Level III	4/4
Jarus et al., 2011 [25]	Waitlist control trials	Quantitative nonrandomization controlled	Level II	3/4
Leland et al., 2016 [26]	RCT	Quantitative randomization controlled (trials)	Level I	3/4
Schoen et al., 2017 [27]	A quasi-experimental, single-group, pretest/posttest design	Quantitative descriptive	Level III	2/4
Wen et al., 2017 [28]	Mixed-methods pilot study	Mixed methods	Level IV	1/4
Wolfhope et al., 2016	Mixed-methods pilot study	Mixed methods	Level IV	2/4
Wooster et al., 2015 [30]	A pretest-posttest, one group design	Quantitative descriptive	Level III	2/4

**Table 2:** Risk of bias table for the RCTs.

Author, year	Selection bias		Performance bias	Detection bias		Attrition bias		Reporting bias
	Random sequence generation	Allocation concealment	Blinding of participants	Patient-reported outcomes	All-cause mortality	Short-term	Long-term	Selective reporting
Farrehi et al., 2016 [21]	+	+	+	+	?	?	?	+
Gutman et al., 2016 [23]	?	?	?	?	?	+	?	+
Leland et al., 2016 [26]	+	+	+	?	?	+	+	+

*Note.* Categories for risk of bias are as follows: +: low risk; ?: unclear risk; −: high risk.

**Table 3:** Characteristics of included studies.

Author, year, country	Setting subject	Sample size (*n*), age, sex (M/F)	Level of evidence/clinical study design, inclusion/exclusion criteria (IC/EC)	Type of intervention	Outcome measure	Results
Eakman et al., 2016, USA	Various university campuses, 911 US Military veterans	*n* = 8, age 35.6 ± 7.4, sex 8/0	Level III Single-arm feasibility pilot study IC: post 911 military veteran attending college, service-connected injury, reported sleep difficulties, willing to complete daily diaries EC: diagnosis of epilepsy or bipolar disorder	Two months of sleep intervention: restoring effective sleep tranquility (REST) program 7 group sessions 8 individual sessions CBTi (i) Sleep restriction (ii) Stimulus control Sleep hygiene	(i) Sleep problems index II of the medical outcomes study sleep measure (MOS-sleep) (ii) Patient-reported outcomes measurement information system-sleep disturbance (PROMIS-SD) (iii) Pittsburgh Sleep Quality Index (PSQI) (iv) Dysfunctional Beliefs and Attitudes about Sleep Scale (DBAS) (v) Patient-reported outcomes measurement information system-ability to participate in social roles and activities (PROMIS-AP) (vi) Patient-reported outcomes measurement information system-satisfaction with participation in social roles (PROMIS-SP) (vii) Patient-reported outcomes measurement information system-pain interference (PROMIS-PI) (viii) Canadian Occupational Performance Measure (COPM)	(i) Reduced sleep difficulties (*t* = 3.29, *p* = 0.02) (ii) Reduced nightmares (*t* = 2.79, *p* = 0.03) (iii) Fewer dysfunctional sleep-related beliefs (*t* = 3.63, *p* = 0.01) (iv) Greater ability to participate in social roles (*t* = −2.86, *p* = .03) Trends toward improved satisfaction with participation and reduced pain interference

Farrehi et al., 2016, USA [21]	Hospital, aged 18–75	*n* = 120, age 56.22 ± 11.41	Level I RCT	(i) Intervention group: occupational therapy sleep tool intervention (eye mask, ear plugs, and white noise machine) sleep education on environment control (ii) Control group: sleep education	(i) COPM (ii) FIM (iii) Patient-Reported Outcome Measurement Information System Survey: fatigue, physical functioning, sleep disturbance, wake disturbance (iv) Brief Pain Inventory (short form) (v) Pain reduction associated with sleep deprivation	(i) Significant reduction of fatigue scores over 3 days, compared with controls (*p* = 0.02) (ii) Trend toward improvement in sleep disturbance, sleep-related impairment, physical functioning, pain severity, or paint interference (*p*>0.1) (iii) No difference in length of stay (*p* = 0.9) or use of opioids (*p* = 0.7)

Gee et al., 2017, USA [22]	Autism spectrum disorder	*n* = 4, age 3–6 years old	Level V An ABA single-subject design IC: children had a diagnosis of ASD, present with sleep problem, sensory over responsibility	Use of weighted blankets	(i) Sleep quality (ii) Time to fall asleep (iii) Sleep duration (iv) Behavioral ratings on waking	(i) Moderate improvement of the measured constructs related to sleep quality (ii) Increased in total amount of sleep per night (iii) Decrease in time to fall asleep (iv) Sleep between 1 and 3 hours a night more as a result of the weighted blanket

Gutman et al., 2016, USA [23]	Community living, adults aged 25–65	*n* = 29, age 43.2 ± 12.2, sex 9/20	Level I RCT IC: poor sleep for 2 months, agreed to follow sleep hygiene for 3 weeks EC: taking sleeping pills, suffered from pain, medical diagnosis causing sleep disruption, pets or family members causing sleep disruption, pregnant or smokers	Three weeks of sleep intervention of the following: (i) Dreampad pillow (ii) iRest meditation (iii) Sleep hygiene	(i) General Sleep Disturbance Scale (ii) Pittsburgh Sleep Quality Index (iii) Actigraph accelerometer (iv) Sleep diary	(i) iRest meditation group experienced statistically more time asleep than both the Dreampad pillow (*p* < 0.006) and sleep hygiene groups (*p* < 0.03) (ii) Dreampad pillow group experienced statistically fewer nighttime awakenings than iRest meditation (*p* < 0.04) and sleep hygiene groups (*p* < 0.004) (iii) No difference was found between groups in perceived sleep quality, length of time needed to fall asleep, or next-day fatigue level

Heidt et al., 2016, USA [24]	Hospital, aged 18–75	*n* = 52, age 57.5 ± 9.9, sex 29/23	Level III Experimental study design with single sample and pre-post testing	(i) Simple sleep-enhancing education (ii) Sleep-enhancingtools	(i) Patient-Reported Outcome Measurement Information System Survey: fatigue, physical functioning, sleep disturbance, wake disturbance	(i) Significant improvement in fatigue (*t* = 5.5, *p* < 0.001), sleep disturbance (*t* = 3.9, *p* < 0.001), and wake disturbance (*t* = 3.8, *p* < 0.001) (ii) No significant improvement in the physical function aspect (*p* = 0.1)

Jarus et al., 2011, Canada [25]	Meir Medical Center, preterm infants	*n* = 32, postmenstrual age (days) 30.37 ± 2.57, sex 12/20	Level II RCT IC: birth weight less than 1750 g, stable in room air EC: major congenital anomalies or major neurological illness, using medication affects the infant's sleep-wake cycle	(i) Alternate position every 3-4 hours after feedings	(i) Actigraph measurement (ii) Naturalistic observations of newborn behavior (NONB)	(i) In the prone position, there were more approach reactions than withdrawal reactions (*p* < 0.001) while in the supine position (ii) In the prone position, more patterns were observed as opposed to more awake patterns

Leland et al., 2016, USA [26]	Various elderly community centers, age > 65	*n* = 217, age 74.2 ± 7.7, sex 65/141	Level I RCT	(i) Occupation-basedintervention	(i) SF 36 (ii) Center for Epidemiologic Studies Depression Scale-Revised (iii) Sleep time (iv) Napping time	(i) The average time sleeping was 8.2 hours daily with SD 1.7 (ii) 29% of participants reported daytime napping at baseline, 36% of whom no longer napped at follow-up Among participants who stopped napping, those who received an occupation-based intervention replaced napping time with nighttime sleep, and those who did not receive an intervention experienced a net loss of total sleep (*p* < 0.05)

Schoen et al., 2017, USA [27]	South Shore Therapies and Knippenberg, Patterson, Langley, & Associates, children with autism spectrum disorder	*n* = 15	Level III A quasi-experimental, single-group, pretest/posttest design IC: parents reported moderate to severe sleep disturbance EC: had stressful life circumstance that could account for new onset sleep difficulties, medical or psychiatric illness, medication known to cause insomnia or sedation, receiving medication or CBT for sleep disorder, could not comply with sleep diary or use of pillow	(i) iLs Dreampad pillow	(i) A sleep diary documented average sleep duration and average time to fall asleep during the preintervention phase and the last 2 weeks of the treatment phase (ii) The Children's Sleep Habits Questionnaire (CSHQ) (iii) The Pediatric Quality of Life Inventory (PedQL) (iv) The Parental Concerns Questionnaire (PCQ) (v) The Swanson, Nolan, and Pelham (SNAP-IV)	(i) Procedures were acceptable and feasible for families. All measures were sensitive to change. Children with ASD demonstrated significant change in sleep duration (*t* = −3.01, *p* < 0.003) and time needed to fall asleep (*t* = −2.83, *p* < 0.005) from pretest to intervention (ii) Improvements were noted in autism-related behaviors, attention (*t* = −2.63, *p* < 0.009), and quality of life (*t* = −2.94, *p* < 0.003), SNAP-IV (*t* = −2.44, *p* < 0.015); parent satisfaction was high

Wen et al., 2017, USA [28]	Traumatic brain injury	*n* = 2, age 31	Level IV Mixed-methods pilot study IC: diagnosis of chronic TBI (6-month postinjury), ability to stand/move, ability to follow a 3-step command, ability to read/speak English EC: neurological conditions like bipolar disorder and attention deficit hyperactive disorder	(i) Yoga (ii) Breathing exercise	(i) Pittsburgh Sleep Quality Index (ii) Neuropathy Pain Scale (iii) Behavior Rating Inventory of Executive Function (iv) Beck Depression Inventory	(i) One participant showed 25% reduction in depressive symptoms, and other improvements were found in the inhibition and emotional control scales of the BRIEF

Wolfhope et al., 2016, USA [29]	Saint Francis University, autism spectrum disorder	*n* = 2, age 3–6	Level IV A preexperimental single-case design and followed the OXO research design	(i) iLs Dreampad mini	(i) Self-created questionnaire (ii) FitBit Flex	(i) Increase in the number of hours of sleep received per night (ii) Increase in observed attention and focus (iii) Decrease in meltdowns

Wooster et al., 2015, USA [30]	Various community settings, children with autism spectrum disorder		Level III A pretest-posttest, one group design	(i) Occupational therapy-basedparent educational program (ii) Sensory calming strategies, sleep hygiene xroutines, sleep schedules, bedtime routines, environmental modification, faded bedtime practices, and bedtime pass techniques	(i) Knowledge-basedpretest-posttest was designed and administered before and after the educational program (ii) Children's Sleep Habits Questionnaire (CSHQ): bedtime resistance, sleep anxiety, sleep onset delay, sleep duration, night waking, daytime sleepiness, sleep-disordered breathing, and parasomnias	(i) Significant increase in parental knowledge (*p* = 0.003) on the basis of the occupational therapy educational program provided
